# Petri Net based modeling and analysis for improved resource utilization in cloud computing

**DOI:** 10.7717/peerj-cs.351

**Published:** 2021-02-08

**Authors:** Muhammad Rizwan Ali, Farooq Ahmad, Muhammad Hasanain Chaudary, Zuhaib Ashfaq Khan, Mohammed A. Alqahtani, Jehad Saad Alqurni, Zahid Ullah, Wasim Ullah Khan

**Affiliations:** 1 Department of Computer Science, Western Norway University of Applied Sciences, Bergen, Norway; 2 Department of Computer Science, COMSATS University Islamabad, Lahore Campus, Lahore, Pakistan; 3 Department of Electrical & Computer Engineering, COMSATS University Islamabad, Attock Campus, Attock, Pakistan; 4 Department of Computer Information Systems, College of Computer Science and Information Technology, Imam Abdulrahman Bin Faisal University, Dammam, Saudi Arabia; 5 Department of Educational Technology, College of Education, Imam Abdulrahman Bin Faisal University, Dammam, Saudi Arabia; 6 Department of Information Systems, Faculty of Computing and Information Technology, King Abdulaziz University, Jeddah, Saudi Arabia; 7 School of Electrical Engineering and Automation, Wuhan University, Wuhan, China

**Keywords:** Cloud computing, Replication, Colored Petri net, Formal analysis

## Abstract

The cloud is a shared pool of systems that provides multiple resources through the Internet, users can access a lot of computing power using their computer. However, with the strong migration rate of multiple applications towards the cloud, more disks and servers are required to store huge data. Most of the cloud storage service providers are replicating full copies of data over multiple data centers to ensure data availability. Further, the replication is not only a costly process but also a wastage of energy resources. Furthermore, erasure codes reduce the storage cost by splitting data in *n* chunks and storing these chunks into *n + k* different data centers, to tolerate *k* failures. Moreover, it also needs extra computation cost to regenerate the data object. Cache-A Replica On Modification (CAROM) is a hybrid file system that gets combined benefits from both the replication and erasure codes to reduce access latency and bandwidth consumption. However, in the literature, no formal analysis of CAROM is available which can validate its performance. To address this issue, this research firstly presents a colored Petri net based formal model of CAROM. The research proceeds by presenting a formal analysis and simulation to validate the performance of the proposed system. This paper contributes towards the utilization of resources in clouds by presenting a comprehensive formal analysis of CAROM.

## Introduction

Cloud computing is an emerging paradigm of information technology. Moreover, cloud computing is an IT criterion that provides universal access to shared pools of system resources through the Internet. The resources can be provided on demand on pay or in the form of a subscription. With Internet access growth, cloud computing is emerging in the industry, academia, and society. Due to a large number of resources, the cloud uses virtualization for resource management. Further, clouds need to stimulate data centers’ design so that data can be readily available to users anywhere in the world (Buyya et al., 2009).

### Services

There are four different services in cloud computing.

#### Software as a Service

Software as a Service (SaaS) is a multi-tenant platform that enables cloud users to deploy their applications to the hosting environment. Further, it supports different cloud applications in a single logical environment to achieve optimization in terms of speed, security, availability, scalability, and economy (Dillon, Wu & Chang, 2010).

#### Platform as a Service

Platform as a Service (PaaS) facilitates the cloud user to organize, develop and manage various applications through a complete “software development lifecycle”. Further, it also eliminates the requirement of an organization to traditionally build and maintain the infrastructure, to develop applications (Sajid & Raza, 2013). By using SaaS, cloud users can host different applications while PaaS offers a platform to develop different applications (Dillon, Wu & Chang, 2010; Sajid & Raza, 2013).

#### Infrastructure as a Service

It offers direct access to resources such as storage, computer, and network resources used for processing (Dillon, Wu & Chang, 2010). Infrastructure as a Service (IaaS) sets up an independent virtual machine (VM) to transform the architecture of the application so that multiple copies can be executed on a single machine. Moreover, it provides access to the infrastructure and delivers additional storage for network bandwidth of the corporate web servers and data backups. An important feature of IaaS is that extensive computing can also be switched on, which previously was only accessible to people with the facility of high power computers.

#### Database as a Service

Database as a Service (DaaS) is a self-service cloud computing model. In DaaS, user request database services and access to the resources. DaaS provides a shared, consolidated program to provide database services on a self-service model (Mateljan, Čišić & Ogrizović, 2010).

### Deployment models

Based on environmental parameters including openness, storage capacity and proprietorship of the deployment infrastructure, one can choose a deployment model from the types of cloud deployment models given below. The following are the types of cloud computing available in the literature.

#### Public cloud

Generally, public clouds may be owned and managed by academic or government organizations and it is used by common users and the public. In the traditional regular sense, in public cloud sources, the internet is delivered dynamically and based on self-service via the Internet by an external supplier who shares resources (Ahmed et al., 2012). Moreover, security issues occur in such types of clouds and are more prone to attack. That is why the user has access to the public cloud via the correct validations (Sajid & Raza, 2013).

#### Private cloud

Such kind of infrastructure only works for a specific organization while off-premise private cloud is used by one company and the infrastructure is implemented by another company (Ahmed et al., 2012). There is no restriction of network bandwidth, security risks, and legal requirements in a private cloud, and data is managed within the organization, which is not permitted in a public cloud (Kamboj & Ghumman, 2016).

#### Hybrid cloud

It is a combination of two or more separate cloud infrastructures (public or private) and forms another type of cloud, the so-called hybrid cloud. This concept is also known as cloud bursting where several integrated cloud infrastructures remain unique entities (Mell & Grance, 2011). Hybrid cloud facilitates organizations to shift overflow traffic to the public cloud to prevent service interruption.

#### Federated cloud

To handle the site failure, cloud infrastructure providers have established different data centers at different geographic locations to ensure reliability. However, this approach has many shortcomings, one problem is that the cloud users may find it difficult to know which remote location is best for their application to host. Cloud service providers have a finite capacity and it is difficult for a cloud infrastructure provider to set up different data centers at different geographic locations. This is why different providers of cloud services fall under one umbrella and form a federated cloud (Varghese & Buyya, 2018). In times of work overload, cloud federation offers the opportunity to avail available computational, cost-effective, on-demand, and reliable storage options to other cloud service providers (Buyya, Ranjan & Calheiros, 2010). For example, an EU-based EGI federated cloud shares 300 data centers with 20 cloud providers.

### Issues

Current data centers are hosting multiple applications having time latency from a few seconds to multiple hours (Patterson, 2008). The main focus of Cloud computing is to provide a performance guarantee and to take care of data privacy. With the high growth rate of data on the Cloud, more massive servers’ need is rising day by day. Demand for higher performance is being fulfilled by replicating data in multiple data centers worldwide without thinking about energy consumption. Further, on average, every data center utilizes as much energy as 25,000 households. Data centers are costly and unfavorable for the environment, as they emit more carbon than both Argentina and the Netherlands (Patterson, 2008).

### Need of cache-a replica on modification

**Figure 1:**
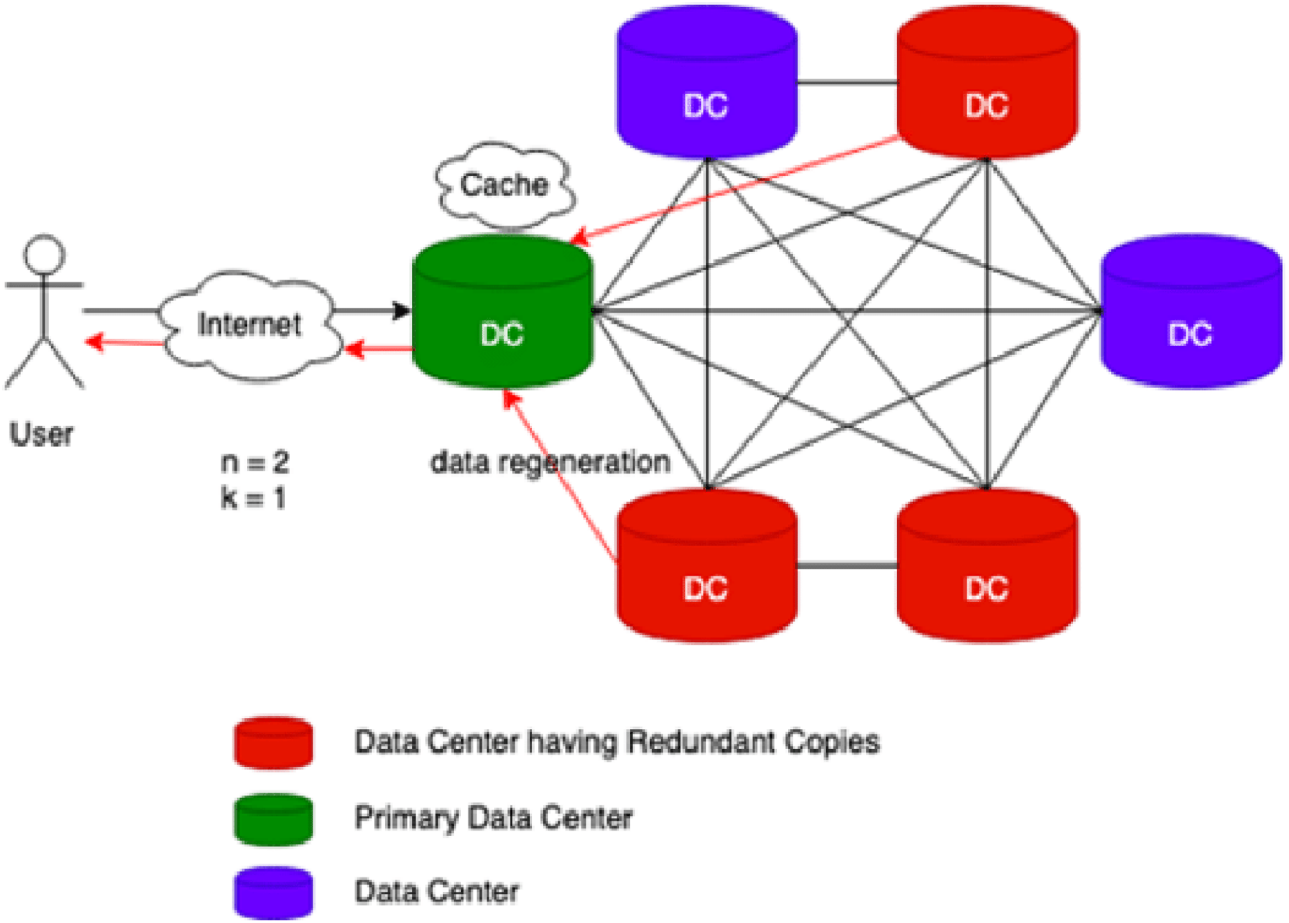
CAROM process flow.

Cache-A Replica On Modification (CAROM) is a hybrid cloud file system that merges the benefits of both replication and erasure codes. Figure 1 reflects the process flow of CAROM. CAROM has a cache at each data center. Cache points out the local access, and every data center performs as a primary data center. The data object which is frequently accessed is stored in the cache to avoid the extra computational cost. In contrast, those objects that are accessed rarely are divided into m data chunks. Further, distribute them among *n + k* data nodes, tolerate *k* failures, and take the storage cost to a minimum and make the data center environment friendly (Ma et al., 2013).

### Contribution of research

Formal methods are mathematical methods used to model or specify any system. Petri net provides strong mathematical and graphical representations to incorporate concurrency, sequential execution, conflicts, determinism, resource sharing, timing information, communication, synchronization, and distribution in the underlying system. This paper's primary goal is to develop a data scheduling model based on colored Petri net (CPN), which utilizes CAROM to reduce storage cost and bandwidth latency. Statistical analysis is provided to elucidate the performance of the model. Simulation is performed, and verification is also presented of the proposed model.

The rest of the article is organized as follows: “Related Work” presents related work. “Colored Petri Nets” presents basic terminology, notations, and graphical convention about Petri Nets. “Formal Model of CAROM” presents the formal modeling of the CAROM based data scheduling framework. “Simulation” presents a formal analysis of the developed model. “Analysis” ****presents the simulations, its results, and the discussion on it. “Conclusion” ****concludes our work and gives final thoughts about the strengths and weaknesses of our approach.

## Related work

In the cloud, resource scheduling is a challenging field (Mathew, Sekaran & Jose, 2014). Magnificent work has been done in resource scheduling in the cloud. Some approaches are relevant to resource scheduling in the cloud. This approach’s immediate attention is to optimize time performance, like completion time, total delay, and response time (Mathew, Sekaran & Jose, 2014). Zhan et al. (2015) provides a detailed survey of cloud computing. Ant colony optimization algorithm for scheduling tasks according to budget is presented in Zuo et al. (2015). In Adil et al. (2015), a heuristic algorithm is proposed for task scheduling. Kumar & Verma (2012) presents a genetic algorithm to schedule independent tasks. In Mateescu, Gentzsch & Ribbens (2011), another genetic algorithm is presented that improves the makespan of resources. The authors of De Assunção, Di Costanzo & Buyya (2010) proposed an architecture that provides a platform for scientifically, high performance (HPC) applications. The cornerstone of the proposed architecture is the Elastic cluster, which expands the hybrid cloud environment (De Assunção, Di Costanzo & Buyya, 2010). Researchers in Mastelic et al. (2015) analyzed the assessment between performance and usage costs of various facilities algorithms for using resources from the cloud to expand a cluster capacity. The authors in Javadi, Abawajy & Buyya (2012) propose non-disruptive source facilities policies for hybrid cloud environments that they have evaluated using a model-based simulation instead of our real case study performance evaluation. Researchers in Mattess, Vecchiola & Buyya (2010) present a facility algorithm for expanding cluster capacity with Amazon EC2 Spot Organizations. Research work in Yuan et al. (2017) provides a profit maximization model for private proposals cloud providers using the temporal variation of prices in a hybrid cloud. Although they are similar to many others, they take time, and data and networks’ costs are negligible.

However, all the algorithms in the literature were limited to static resources only. With the revolution of cloud computing, the number of data servers is increasing across the world. The construction of the data center is not only cost-effective but also not in favor of the environment. Much focus is given to energy-optimized resource scheduling in cloud computing. The researcher has proposed an aware energy model in the form of directed acyclic graphs in Gan, Huang & Gao (2010). In Zhao et al. (2016), two fitness functions are defined: job completion time and energy.

A researcher in Shen et al. (2017) proposed a resource allocation technique that allocates resources to virtual machines taking care of energy. DVFS method has been presented in Hosseinimotlagh, Khunjush & Samadzadeh (2015), which schedules a single task and takes care of the voltage supply. One researcher in Wu, Chang & Chan (2014) has presented a virtual machine scheduling algorithm that achieves energy optimization and reduces host temperature. In Mhedheb et al. (2013), a method is presented to reduce both network and server power. Research work in Xia et al. (2015) scaled the voltage to reduce energy costs. Scaled processor utilization and resource consolidation has been presented in Lee & Zomaya (2012) for energy optimization.

All these methods focus on reducing the cost of energy without the care of job completion time. In Beloglazov, Abawajy & Buyya (2012), the researcher proposed energy-aware mapping of VMs to cloud servers as a problem with bin-packing, independent of the types of workload. Klein et al. (2014) presented a framework of brownout for energy optimization. All users have to bear either time latency or cost on a cloud file system.

## Colored petri nets

Petri nets are bipartite directed graphs with the power of behavioral analysis of the modeled system through it. CPN is a mathematical technique used for modeling parallel systems and graphical analysis of their characteristics (Jensen, 2013; Milner, 1997; Ullman, 1998). CPN is the combination of Petri Net and Standard ML (Emerson & Sistla, 1996; Virendra et al., 2005). CPN allows defining some user-defined data types along with some standard declarations. It is a general-purpose modeling language and has the power to model parallel systems and analyze their performance. Formal Definition of CPN is presented below (Jensen & Kristensen, 2009):

A *net* is a tuple *N* = (*P*, *T*, *A*, *Σ*, *C*, *N*, *E*, *G*, *I*) where:

*P* is a set of *places*.
*T* is a set of *transitions*.
*A* is a set of *arcs* where *P* ∪ *T*=*P* ∩ *A*=*T* ∩ *A*=Ø
*Σ* is a set of color sets
*C* is a color function, that is, *C: P → Σ*
*N* is a node function. It maps A into (*P* × *T*) ∪ (*T* × *P*).
*E* is an arc expression function. It maps each arc *a ∈ A* into the expression *e*.
*G* is a guard function. It maps each transition *t* ∈ *T* to a guard expression *g*. The output of the guard expression should evaluate to Boolean value: true or false.
*I* is an initialization function. It maps each place *p* into an initialization expression *i*.

We can map each place into a multi-set of tokens in CPN through a mapping function called Marking. Initial Marking reflects the initial state of a model. Final Marking represents the final state of the system.

## Formal model of carom

For modeling, high-level architecture and the components of the system are identified in the first phase. After that, the identified components’ interaction points are defined for the smooth implementation of the component-based architecture. Further, a mixture of top-down and bottom-up approaches is adopted in this paper to model the framework. CAROM uses some part of the local storage disk as a cache. Whenever a written request of a new file is received, the complete file is stored in the reserved memory of each DC named as cache.Whenever the cache is near to be filled, the file least recently used is removed from the cache. It is distributed on *n + k* data nodes after dividing into *n* chunks.

**Figure 2:**
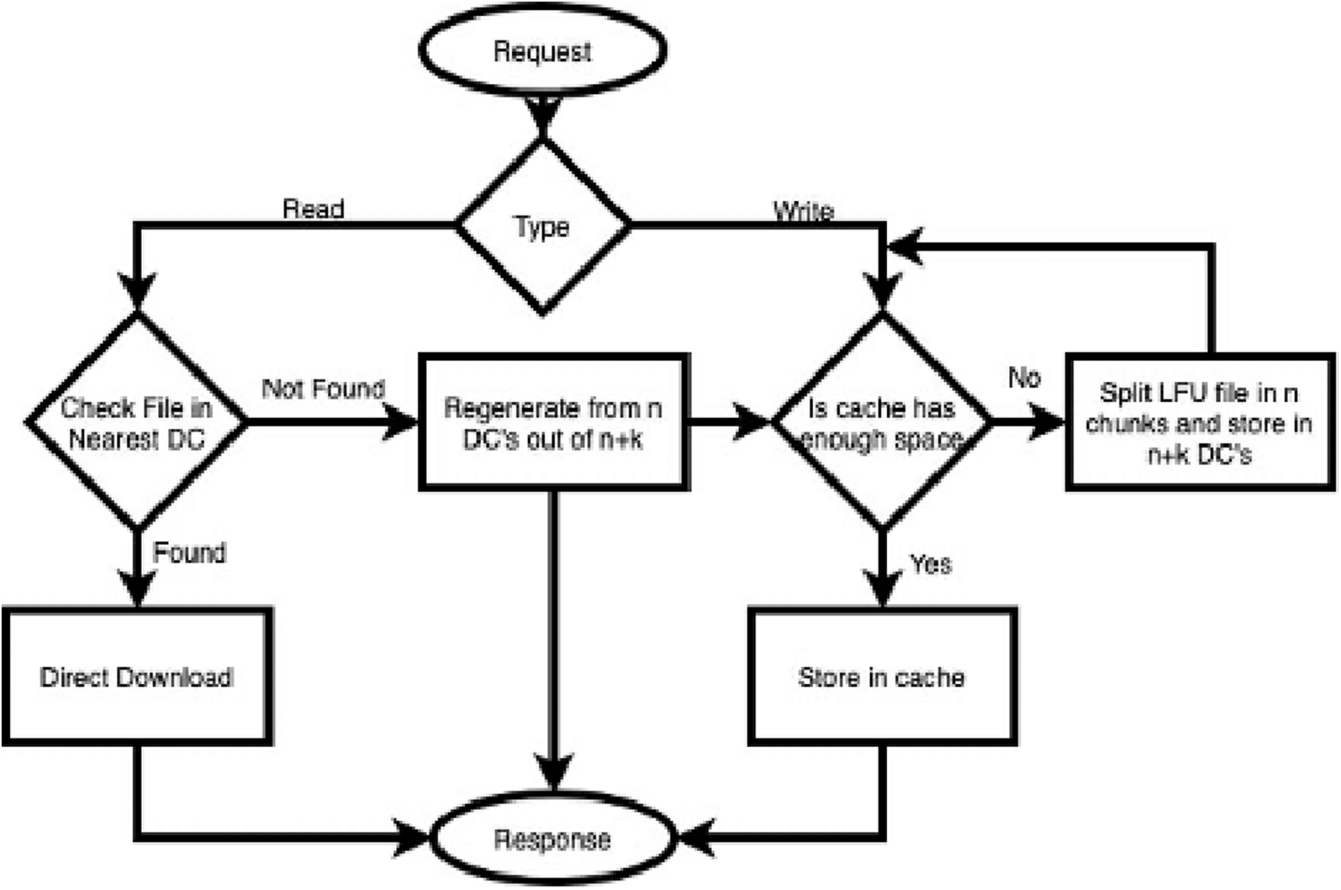
File access process flow of CAROM.

However, suppose a read request for a file is received. In that case, it is checked first in the nearest DC. If it is found, then it is downloaded directly, without any computational cost. Whenever a request of that file is received that is not available in the cache. Data is regenerated from n data nodes out of *n + k* (Ma et al., 2013). The strategy discussed above is presented in the form of a flow chart (see Fig. 2).

### Hierarchical view of model

**Figure 3:**
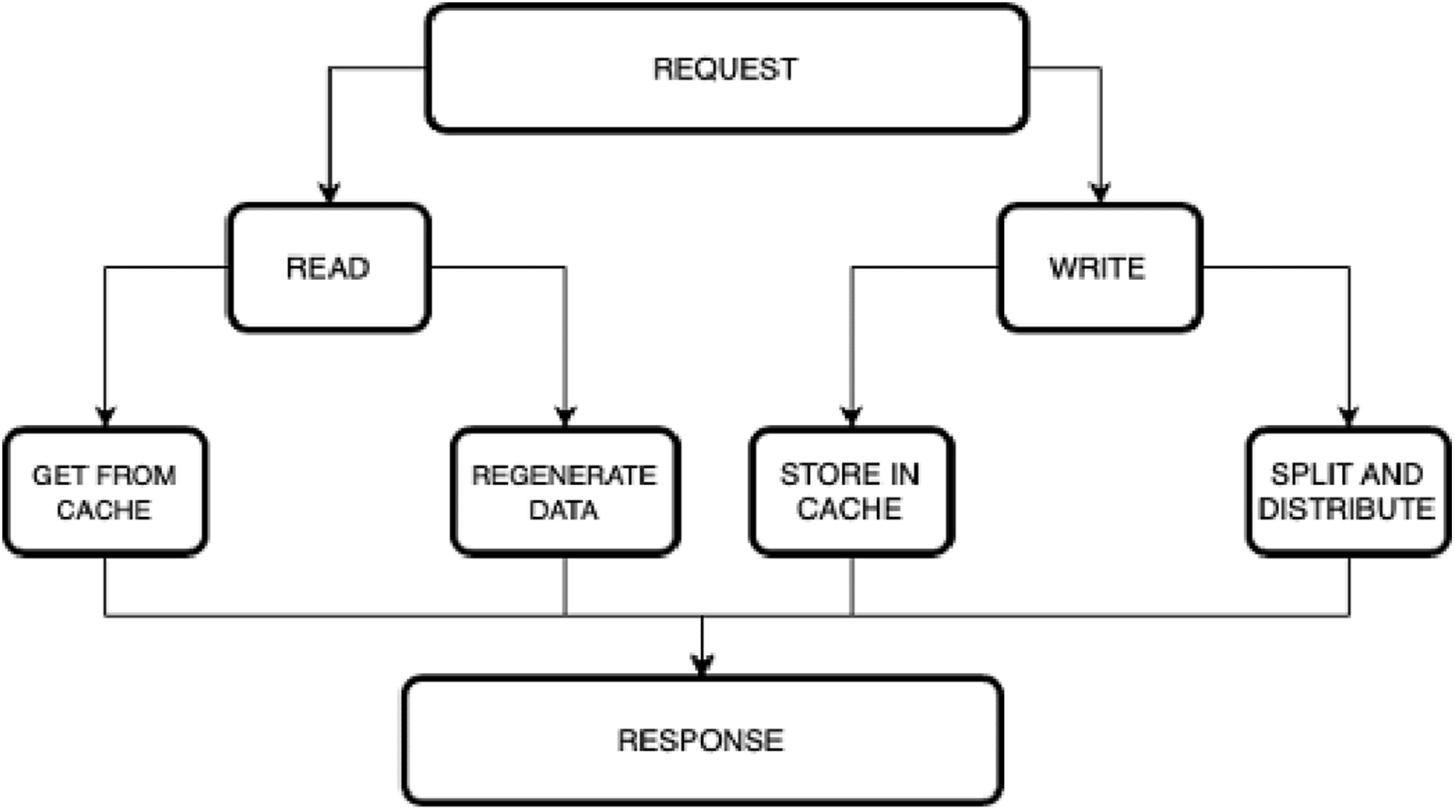
Hierarchical view of the model.

Figure 3 depicts the hierarchical view of the model.

### Colored Petri nets model

**Figure 4:**
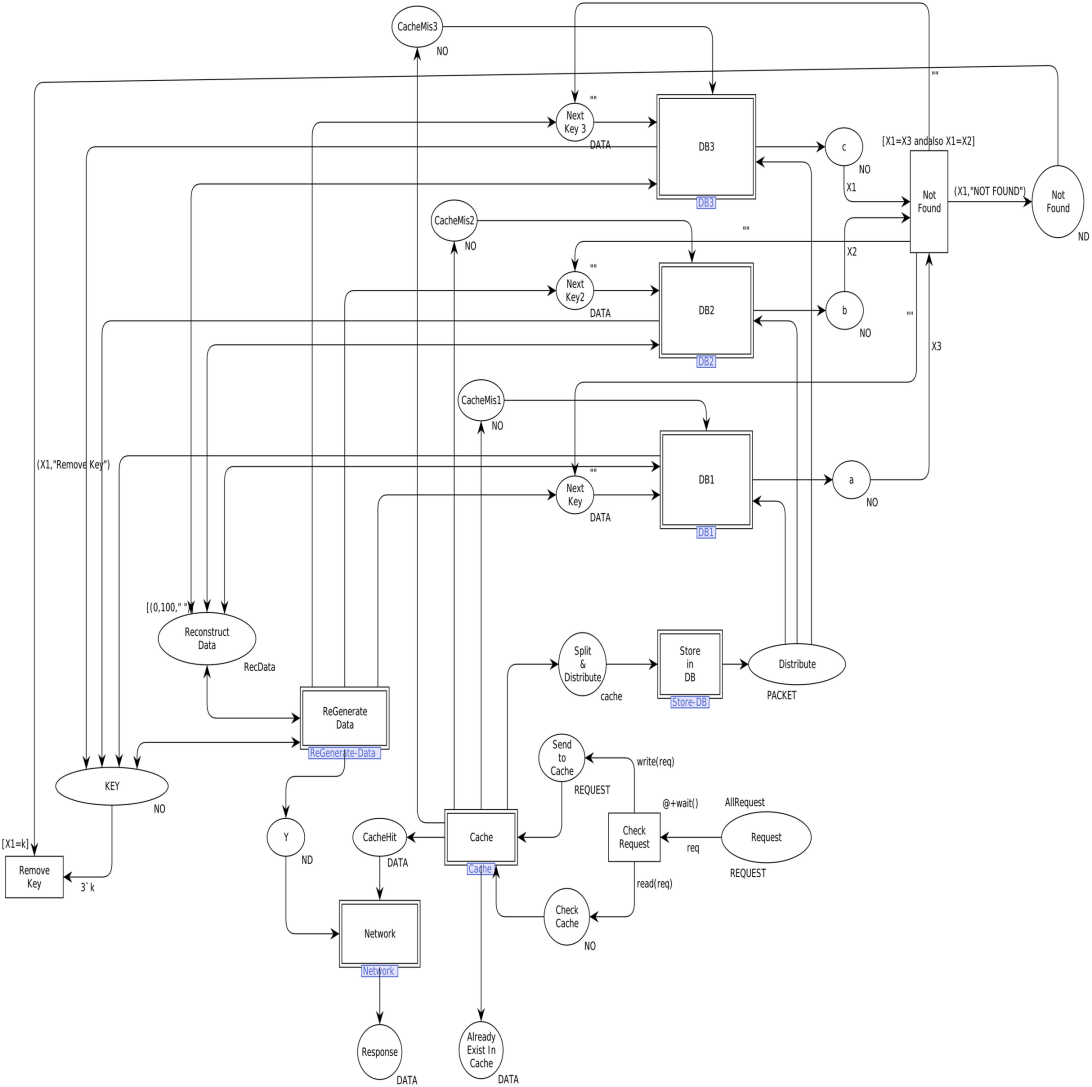
Top level view of proposed scheme.

In order to model the CAROM based framework using CPN, the components *Sender*, *Data Center*, and *Receiver* are developed. *The Data Center* component is further extended to *Cache* and *DataNode* sub-components, as shown in Fig. 3. Table 1 represents the color sets used in the model. As data types, the color sets are mapped to the places of the model given in Fig. 4. For instance, color set *NO*, in the third row of Table 1, is mapped to the place *KEY* while color set *DATA*, in the fourth row of Table 1, is mapped to the place *Next_Key* in the CPN model shown in Fig. 4. Moreover, product type color sets are constructed by taking the cartesian product of the color sets. For instance, the color set *REQUEST* in Table 1 is constructed using color sets *NO, DATA, OP* and *NO*.

**Table 1:** Color sets of the model.

Color set	Defination
colset UNIT = unit;	Unit color set
colset BOOL = bool;	Boolean color set
colset toa = int; closet NO = int;	Integer color sets
colset DATA = string timed;	Timed string color set
colset OP = string timed;	Timed string color set
colset REQUEST = product NO × DATA × OP × NO timed;	Timed product of color set NO of type int, color set DATA of type string, color set OP of type string and color set NO of type int.
colset File= product NO × DATA × toa timed;	Timed product of color set NO of type int, color set DATA of type string and color set toa of type int.
colset ND=product NO × DATA timed;	Timed product of color set NO of type int and color set DATA of type string.
colset RR=product NO × NO timed;	Timed product of color set NO of type int and color set NO of type int.
colset RRL=list RR timed;	Timed list of color set RR.
colset nkd=product NO × NO × DATA;	Product of color set NO of type int, color set NO of type int and color set DATA of type string.
colset RecData = list nkd timed;	Timed list of color set nkd.
colset PACKET = union Data:nkd timed ;	Union of type Data(int*int*string),
colset sendData= product NO × NO × DATA timed;	Timed product of color set NO of type int, color set NO of type int and color set DATA of type string.
colset sendList=list sendData timed;	Timed list of color set sendData.
colset cache = product NO × DATA × NO timed;	Timed product of color set NO of type int, color set DATA of type string and color set NO of type int.
colset CacheList = list cache timed;	Timed list of color set cache.
colset CacheHit= product NO × CacheList timed;	Timed product of a color set NO of type int and color set CacheHit of type list.
colset rcvSplit = product NO × PACKET;	Product of color set NO of type int and color set PACKET of type union.
colset packet=list PACKET timed;	Timed list of color set PACKET of type union.

Table 2 represents the list of variables used in the model. A variable *v* is used in the arc inscription, and *Type[v] ∈ Σ*, to fetch the data from the place. Further, the variables construct arc expression, which is assigned to arc a through arc expression function *E*: *A→EXPRV* while *Type[E(a)] = C(p)MS* where *EXPR* is the set of expressions and *MS* is a multiset. A marking is a function *M* that maps each place *p ∈ P* into a multiset of tokens, that is, *M(p)* ∈ *C(p)MS*. Table 3 shows values (tokens) to represent the initial marking. Arc expressions are evaluated by assigning the values to the variables in the expressions. Further, expressions can be converted into functions to be mapped to arcs. Table 4 represents the functions used in this model.

**Table 2:** Variables of the model.

Variable	Defination
var p:PACKET;	Variables of colour set PACKET.
var pak:packet;	Variable of colour set packet.
var d,data,next:DATA;	Variables of colour set DATA.
var n,n1,id,k,k1,X1,X2,X3:NO;	Variables of colour set NO.
var cl:CacheList;	Variables of colour set CacheList.
var sl:SendList;	Variable of colour set SendList.
var c:cache;	Variable of colour set cache.
var rd,sd:RecData;	Variables of colour set RecData.
var req,e:REQUEST;	Variables of colour set REQUEST.

**Table 3:** Initializations of the model.

Initial marking
val db1=1`[Data(1,1,"C"),Data(2,1,"O"),Data(3,1,"E"),Data(4,1,"P")]@0;
val db2=1`[Data(1,2,"O"), Data(2,2,"U"),Data(3,2,"D"),Data(4,2,"E")]@0;
val db3=1`[Data(1,3,"L"),Data(2,3,"R"),Data(3,3," "), Data(4,3,"T")]@0;
val AllRequest= 1`(1,"COL","WRITE",0)@0 +++ 1`(2,"OUR","WRITE",0)@0+++ 1`(3,"ED ","WRITE",0)@0+++ 1`(1," ","READ",0)@0+++ 1`(2," ","READ",0)@0+++ 2`(4,"PET","WRITE",0)@0+++ 1`(5,"RI ","WRITE",0)@0+++ 1`(5," ","READ",0)@0;

**Table 4:** Functions of the model.

Function	Purpose
fun check(n,k) = if(n>k) then n else k;	Enable transition if first token has greater value
fun wait() = discrete(10,100);	Wait for a random time unit between 10 and 100
fun check1(n,k) = if n=k then k+1 else k;	Increment if both tokens have same value
fun success() = discrete(1,10)<=9;	To check either random number is less than 9
fun success1(n,d) = if success() then 1`(n,d) else empty;	Enable transition if random number is less than 9
fun success2(k) = if success() then 1`k else empty;	Enable transition if random number is less than 9
fun transmit(n,k,d) = if n=k then 1`d else empty;	Enable transition if both tokens have same value
fun transmit1(n,k) = if n>k then 1`k else empty;	Enable transition if first token has greater value
fun read(req:REQUEST) = if #3(req)="READ" then 1`(#1(req)) else empty;	Enable transition with 1st argument of request if 3rd argument of request is “READ”
fun write(req:REQUEST)= if #3(req)="WRITE" then 1`req else empty;	Enable transition with whole request if 3rd argument of request is “WRITE”
fun length [] = 0 | length ( h :: t ) = 1+length t;	Return length of a list
fun cacheMember(req:REQUEST,[])=false | cacheMember(req,(n,d,k)::t)=if(#1(req))=n then true else cacheMember(req,t);	Check either a file exist in cache or not
fun store(req:REQUEST,cl:CacheList) = ifcacheMember (req,cl) then cl else (#1(req),#2(req),#4(req))::cl;	Store a file on cache
fun member (k1,[]) = false | member (k1,(k2,v2,k3)::t) =if k1=k2 then true else member (k1,t);	Check either a token exist in a list or not
fun member2((k,n,d),[])=false| member2((k,n,d),(k1,n1,d1)::t)=if k=k1 andalso n=n1 andalso d=d1 then true else member2((k,n,d),t);	Check either a token exist in a list or not
fun remDup((k,n,d),sd)= if member2((k,n,d),sd) then sd else (k,n,d)::sd;	Remove duplications
fun insert((k,n,d),[])=[(k,n,d)] | insert((k,n,d),(k1,n1,d1)::t)=if n<=n1 then (k,n,d)::(k1,n1,d1)::t else (k1,n1,d1)::insert((k,n,d),t);	Insert in a list
fun insert1((n,d,k),[])=[(n,d,k)] | insert1((n,d,k),(n1,d1,k1)::t)=if k<=k1 then (n,d,k)::(n1,d1,k1)::t else (n1,d1,k1)::insert1((n,d,k),t);	Insert in a list
fun insert3((k,n,d),[])=[(k,n,d)] | insert3((k,n,d),(k1,n1,d1)::t)=if n<n1 then (k,n,d)::(k1,n1,d1)::t else (k1,n1,d1)::insert3((k,n,d),t);	Insert in a list
fun sort[] = [] | sort ( (n,d,k)::t)= insert1((n,d,k),sort t);	Sort with respect to least frequently used
fun sort1[]=[] | sort1((k,n,d)::t) = insert3((k,n,d),sort1 t);	Sort with respect to least frequently used
fun member1(k1,[])=false | member1 (k1,(k,k2,v2)::t)= if k1=k2 then true else member1(k1,t);	Check either a token exist in a list or not
fun recData(k,n1,d,rd)=if member1(n1,rd) then rd else insert((k,n1,d),rd);	Reconstruct data
fun checkDB(k,[])=false | checkDB(k,Data(k1,k2,v)::t) = if k=k1 then true else checkDB(k,t);	Check data in data base
fun recD1(k,Data(k1,k2,v)::t,rd,recData)=if k=k1then recData(k1,k2,v,rd) else recD1(k,t,rd,recData);	Enable transition if data is regenerated
fun found(k,pak,rd,recD1)=if checkDB(k,pak) then recD1(k,pak,rd,recData) else rd;	Enable transition if data is found in data base
fun notFound(k,pak)=if checkDB(k,pak) then empty else 1`k;	Enable transition if data is not found in data base
fun retrieve(k1,[]) = "NOT FOUND" | retrieve (k1,(k2,v2,k3)::t) = if k1=k2 then v2 else retrieve(k1,t);	Retrieve data from data base
fun cacheHit(member,retrieve,k,cl) = if member(k,cl) then 1`retrieve(k,cl) else empty;	Signal to show data is available in cache
fun cacheMis(member,k,cl)= if member(k,cl) then empty else 1`k;	Signal to show data is not available in cache
fun SendTok(k1,k2,k3,k4) = if k3="WRITE" then 3`(k1,k2,k3,k4) else 1`(k1,k2,k3,k4);	Enable either read or write transition
fun updateLife(k,(k1,v1,k2)::t) = if k=k1 then (k1,v1,k2+1) ::t else updateLife(k,t);	Update frequency
fun SplitData(n,d)= let val p1 = packetLength; fun splitdata (n,k,d) = let val d1 = String.size(d) in if d1<=p1 then [(n,k,d)] else (n,k, substring (d,0,p1)):: splitdata(n,k+1,substring(d,p1,d1-p1)) end; in splitdata(n,1,d) end;	Split data
fun Split(k,data) = ( List.map(fn (n,n1,d)=>Data(n,n1,d))(SplitData(k,data)));	Split data in *n+k* chunks

### Main module

We first identified high-level components of the system, and then each component is step-wise refined. For such a purpose, hierarchical colored Petri nets are appropriate formalism to make the model more straightforward and understandable. Figure 4 depicts the top-level view of the model. This is a hierarchical model in which multiple substitution transitions connect with places. A substitution transition has its own definition. Therefore, groups are identified from the detailed Petri net model and converted into substitution transitions. There are twenty places and ten transitions, including seven substitution transitions, named *Cache, Store-DB, DB1, DB2, DB3, ReGenerate-Data* and *Receiver*.

### Cache module

**Figure 5:**
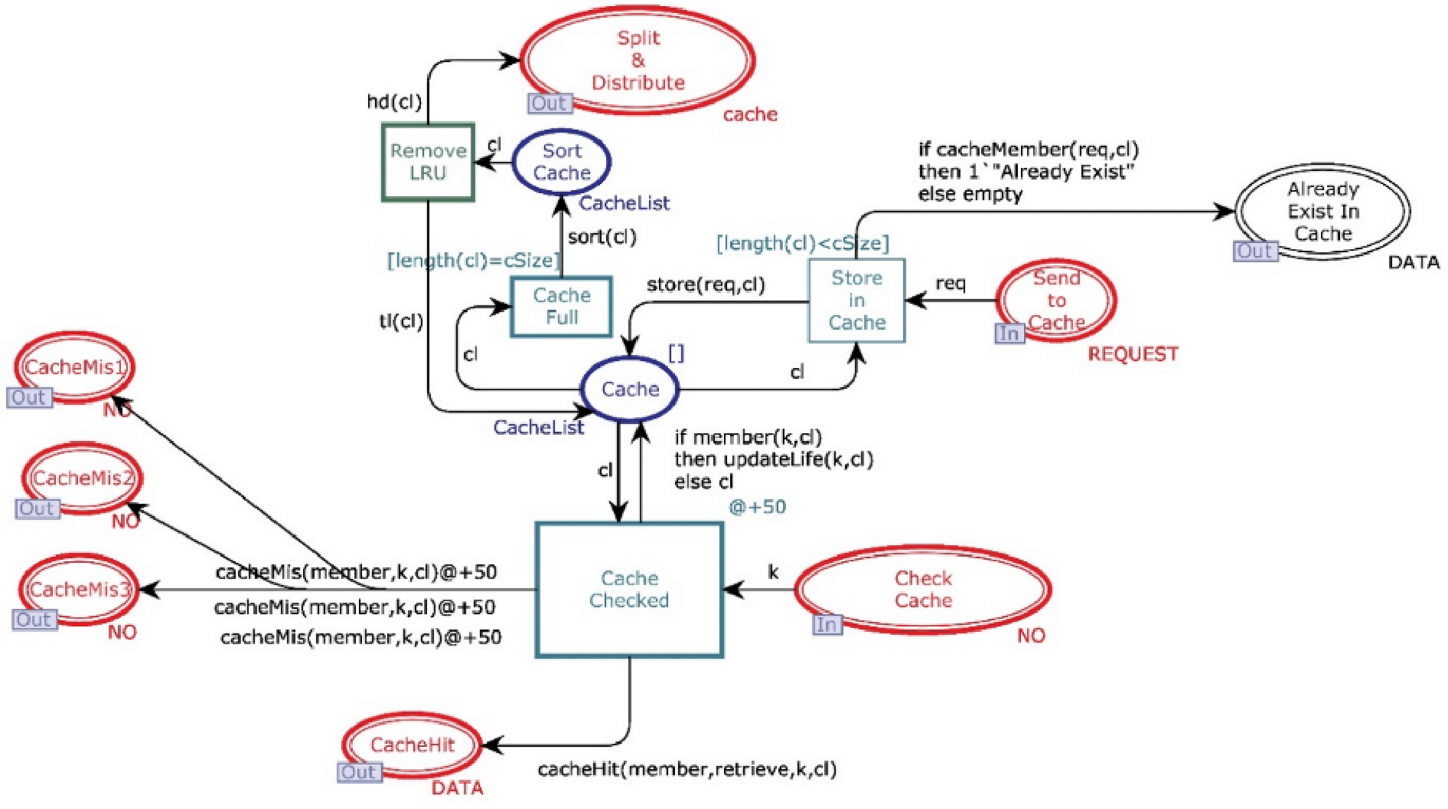
Cache module.

This module aims to decide whether the data will be directly available from cache or reconstruct it from n different data centers. Figure 5 shows the CPN cache module, and it has ten places and four transitions. Two places are in-sockets and six are out-sockets. Whenever a token is added in the place “*Check Cache*” with operation value “READ”, it is sent to transition “*Cache* Checked”, which also receives a “cacheList” from the place “*Cache*”. Function *member* is a Boolean function. It returns true if the key of token coming from the place “*Check Cache*” is found from *cacheList* (see Table 4 for all declared functions). If *member* function returns true, then the function *retrieve* will get the data against key from the cache.

Further, the function sends data to place “*Cache Hit*” and restores that data object in the cache. In contrast, function *updateLife* will increment the value of the life of this object by 1. On the other side, if the function *member* returns false, then the key is sent to all available data centers through “*CacheMiss*”.

Whenever a token is reached in place “*Send to Cache*” with operation value “*WRITE*”, it causes enabling of the transition “*Store_in_Cache*” which can only be fired when the cache is not full. Moreover, if the cache is not full and no data object is found with the same key, then token is sent to place “*Cache*” and inserted on the head of the *cacheList*. However, if the cache is full, then the token waits in place “*Send to Cache*” until the function *sort* arranges the *cacheList* with respect to life of data objects. Further, the data object having the least life is removed from cache, and it is sent to place “*Split & Distribute*”. If the cache is not full but the *cacheList* has a record with the same key, then token will be sent to place “*Already Exist In Cache*” by firing the transition “*Send to Cache*”.

### Store in DB module

**Figure 6:**
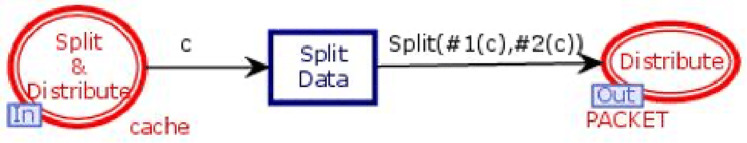
Store in DB module.

Figure 6 shows the Store in DB module of the model. It has two places and one transition. One place is in-socket and one place is out-socket. Whenever the cache is full, the data object with the least life is removed from the cache, and a token is added in the place “Split & Distribute”. This token enables the transition “Split Data”. Then function Split is called, which divides the data value into *n* data chunks. All the n chunks are sent to place “Distribute” for distribution among *n* + *k* databases.

### DB module

**Figure 7:**
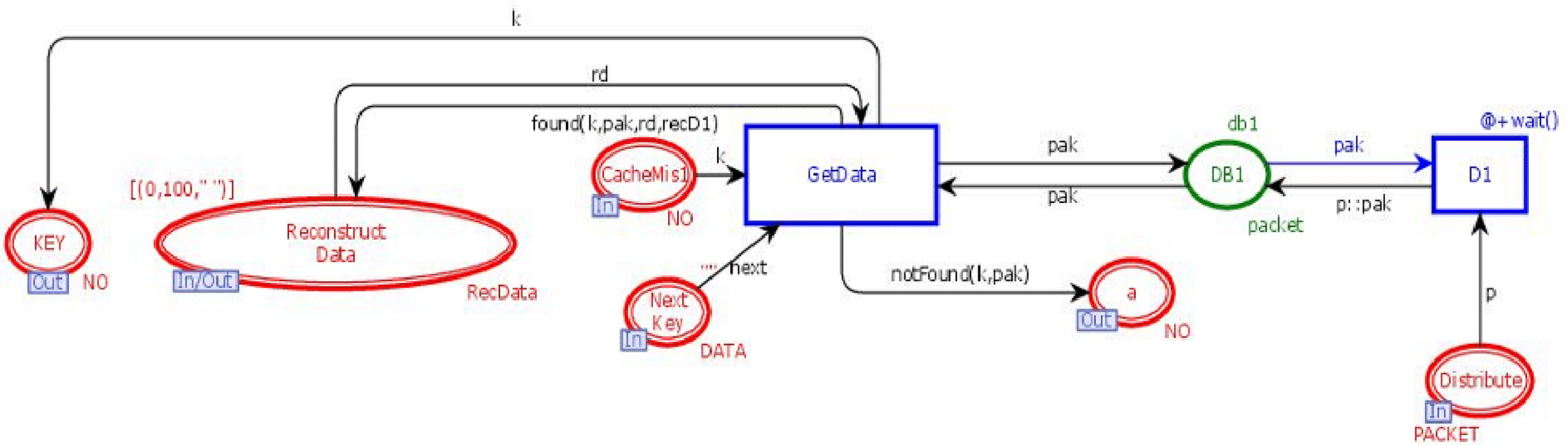
DB module.

This module is to retrieve the n data chunks from *n + k* data centers. DB module contains three in-sockets and two out-sockets. Figure 7 illustrates the DB module of the model. It has seven places and two transitions. Three places are in-sockets, two places are out-sockets and one place is in-out-socket. Whenever a token is reached in place “*Distribute*”, it is stored in the database along with its unique key. Whenever a token having a key is added in the place “*CacheMiss*”, transition “*GetData*” will check the data chunks against that key. If it is found, then the data chunk and its key will be sent to place “*Reconstruct Data*”, which will get *n* data chunks from *n + k* data bases to re-generate the original data with the tolerance of *k* failures.

### Regenerate data module

**Figure 8:**
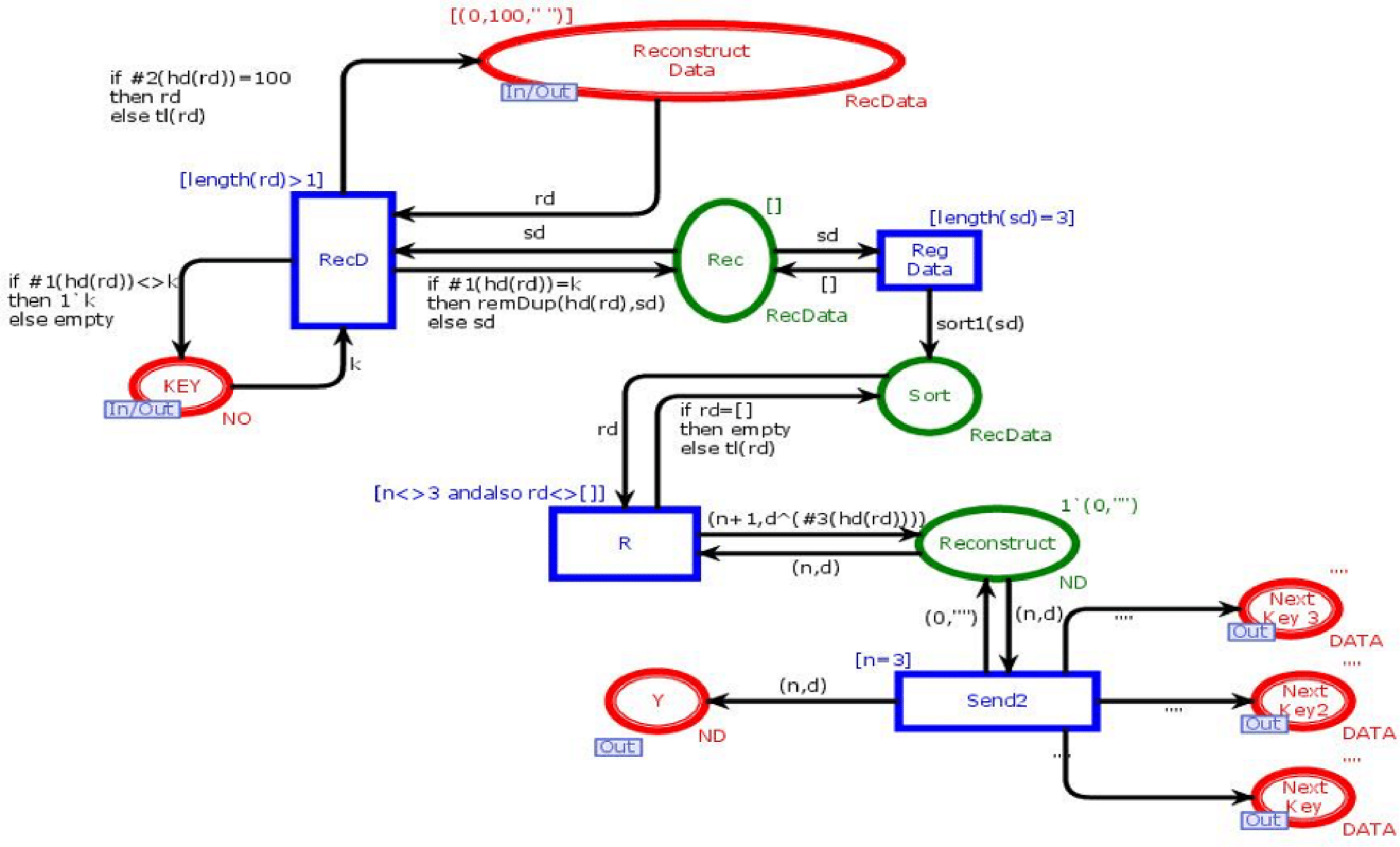
Regenerate data module.

This module is to combine *n* data chunks to reconstruct data into its original form. Figure 8 shows the ReGenerate-Data module of the model. This module has nine places and four transitions. Two places are in-out-sockets and four are out-sockets. In this module, when we need to reconstruct data the place “*Reconstruct_Data*” receives all data chunks against the search key from all available databases. Transition “*RecD*” remains enable until all data chucks move from place “*Reconstruct_Data*”. Then, on arc between the transition “*RecD*” and the place “*Rec*” the function *remDup* (see Table 4) is called, and it removes all the duplications of data chunks. After that, the function *sort1* is called. It sorts data chunks to recontruct the data. The place “*Reconstruct*” holds the token with data in its original form. This place sends data the place “*Reg Data*”, which sends the data towards substitution transition “*Receiver*”.

### Receiver module

**Figure 9:**
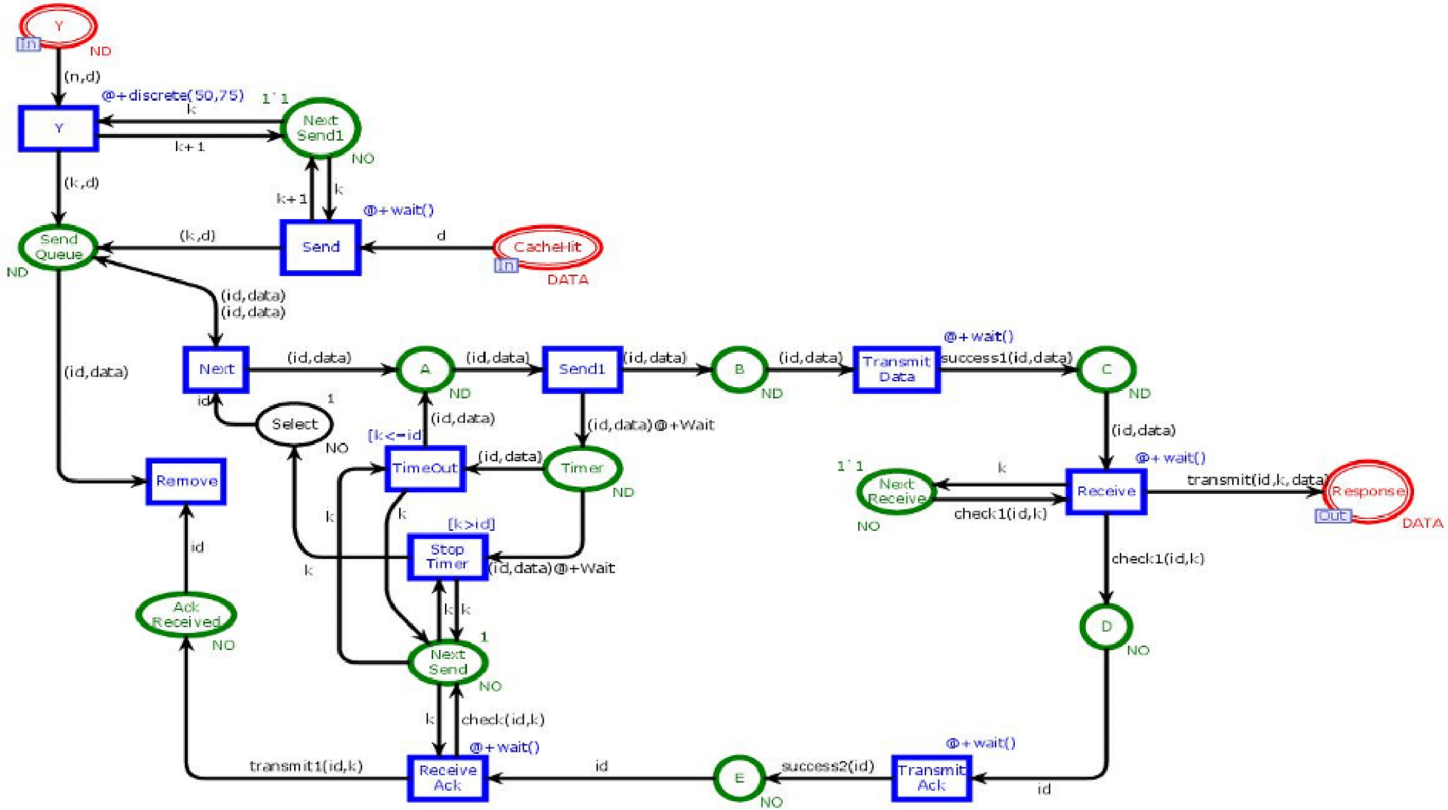
Receiver module.

Figure 9 shows the Receiver module. This module is to ensure that data is ultimately transmitted and received by the user. The receiver module has fifteen places and eleven transiotions. Two places are in-sockets and one is out-socket. In this module, whenever a token is reached in Place *“Y”* or *“CacheHit”* it is sent towards the place *“Send Queue”*. When token from the place *“Send Queue”* enables the transition *“Send1”* then chances of token lost are 90% over a network. If the token is lost, then place *“Timer”* will receive the token. That token will be sent again to avoid the deadlock situation. If the token is sent to place *C* to enable the transition *“Receiver”* then the transition sends data in place *“Response.”* Further, the transition *“TransmitAck”* sends acknowledgment towards the place “*Ack Received”*, which on receiving the token enables the transition *“Remove”* which causes to remove that token from the place *“SendQueue”*.

## Simulation

**Figure 10:**
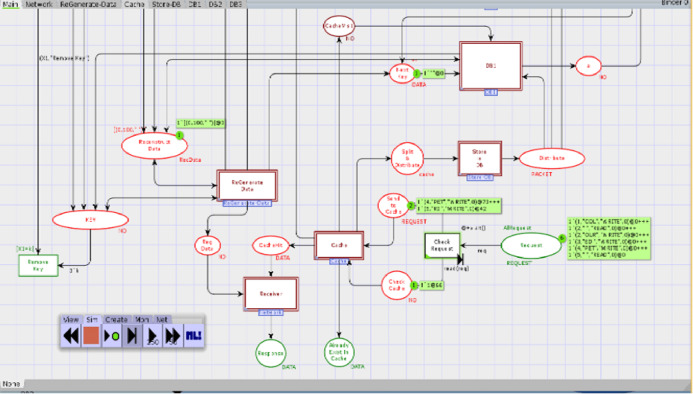
Partial simulation of the module.

Numerous reenactment tools are used to demonstrate and execute a framework, like, process model, SocNetV, Network Workbench. However, it is essential to mention that CPN based formalism supports simulation through CPN Tools. To check the behaviour of the proposed model, we run several manual and ten fully automated simulations of the proposed model with CPN Tools. Figure 10 represents a partial simulation of the model through its intermediate marking (state). In order to get the average completion time of total requests to get both cached and non-cached data, ten simulations are performed (see Table 6). Further, Table 6 shows that simulation 2 gives the high completion time to get cached and non-cached data.

**Table 5:** State space report of the model.

Statistics for O-graph		
**State Space**		
State Space		
Nodes:	56744	
Arcs:	56746	
Secs:	300	
Status:	56746	
Scc Graph		
Nodes:	56744	
Arcs:	56746	
Secs:	2	
**Boundedness Properties**		
**Best Integer Bounds**	Upper	Lower
Cache ′ Cache 1	1	1
DB1 ′ DB1 1	1	1
Main ′ Check_Cache	3	0
Main ′ Request	9	3
Main ′ Send_to_Cache	4	0
Main ′ Response	6	0
Network ′ Next_Receive	1	1
Network ′ Next_Send	1	1
**Liveness Properties**		
Dead Markings		
409 [543, 2369, 6744, 7430, …]		
Dead Transition Instances		
None		
Live Transition Instances		
None		
**Fairness Properties**		
No infinite occurance sequences.		

**Table 6:** Completion time.

Completion time		
	**CACHED**	**NON CACHED**
Simulation 1	114	187
Simulation 2	148	205
Simulation 3	98	151
Simulation 4	107	162
Simulation 5	102	132
Simulation 6	99	144
Simulation 7	133	161
Simulation 8	87	142
Simulation 9	127	174
Simulation 10	118	149

## Analysis

To analyze the performance of the proposed model, we performed the following:

### Verification of model

State-space analysis of the proposed model is performed to monitor the proposed strategy's possible behavior and amend them accordingly (see Table 5).

### Performance analysis

To evaluate the performance of the modeled strategy, average delay, throughput, and average queue lengths are collected by performing ten simulations of the model. For such purpose, monitors are applied on the transitions “*Check Request”, “Cache Checked”, “Split Data”, “Get Data”, “Reg Data”* and *“Receive”* and *places “CacheHit”, “CacheMis1”, “Split”, “Reconstruct Data”* and *“Response”*. Statistical analysis of output data is performed.

Standard behavioral properties and trace patterns generated by our model are analyzed by state space report. Table 5 illustrates the partial statistics generated by state space with 300 s. It reveals that the occurrence Graph (*O-graph*) has 56,744 nodes and 56,744 arcs. Further, these statistics also depict the boundedness properties. The Upper bound shows the maximum number of tokens in a place, while the lower bound shows the minimum number of tokens that can be added to a specific place. It shows that places *Cache, DB1, Next_Receive* and *Next_Send* have both upper and lower bound 1, which means these places always have one token.

However, the upper bound of the place “*Request”* is 9, while it's lower bound is 3. Further, place “*Response”* has upper bound 6 and lower bound equal to zero. It shows that at most 6 requests from place “*Request”* has been fulfilled and stored in place “*Response”*. Liveness properties disclose that there exist 409 dead markings. Dead markings are those markings that have no enabled binding elements. Such dead markings are interpreted as final or terminal markings and not deadlock states of the modeled system. The state-space specifies that the model is partially correct and generated results are correct. Therefore, the state-space analysis conveys that the modeled system behaves according to the requirements and the specifications. Further, the model preserves the properties required for the utilization of storage resources.

**Figure 11:**
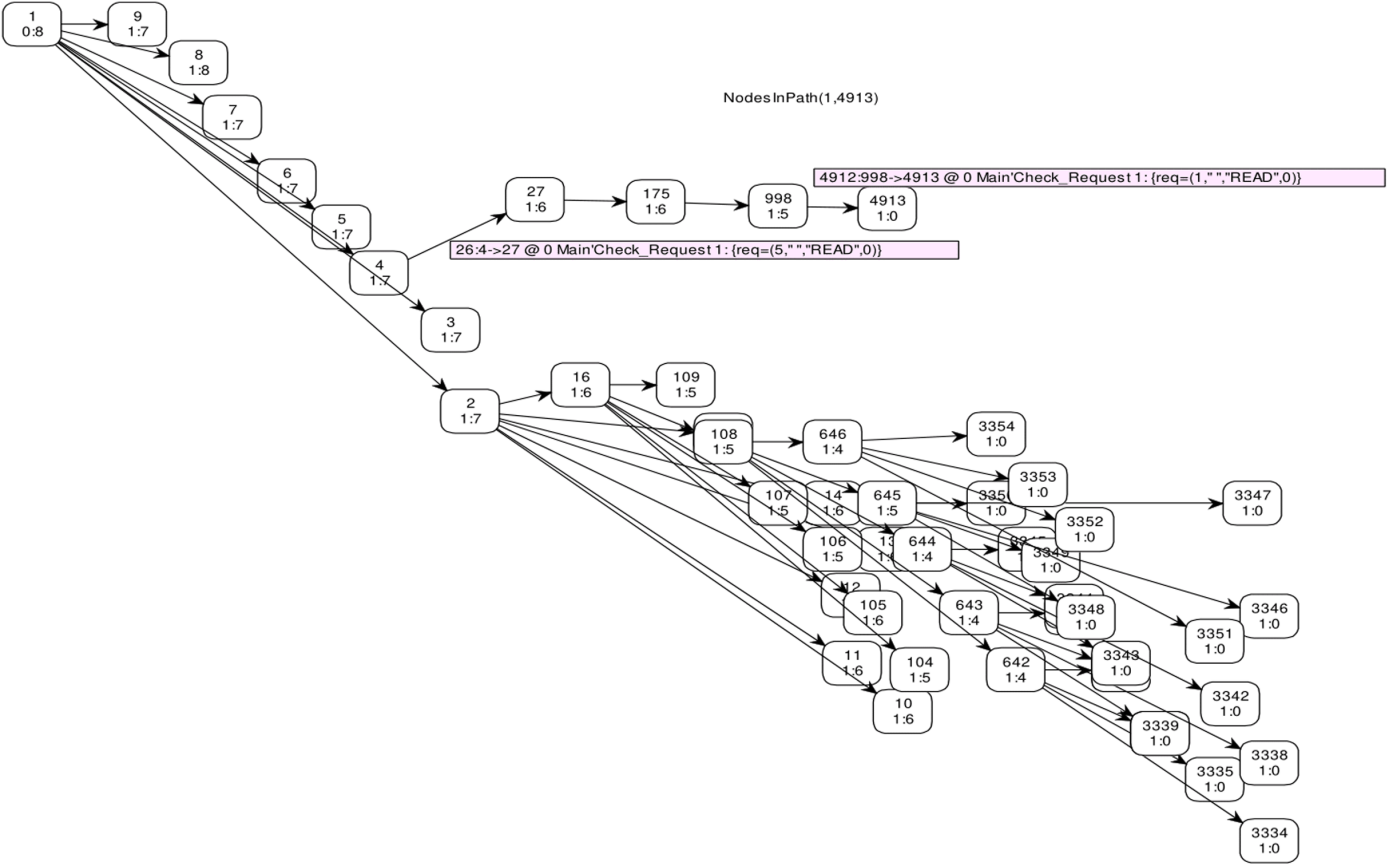
State space graph.

The full state space of CPN has 56,744 nodes and 56,744 arcs, which cannot be depicted in the reachability graph. Therefore, Fig. 11 shows a graphical representation of state space from marking *M* 1–*M* 4913 by skipping some intermediate markings. In CPN Tools, data collection monitors are applied to compute the average completion time. Table 6 depicts the average completion time of total requests to get both cached and non-cached data for ten simulations.

**Figure 12:**
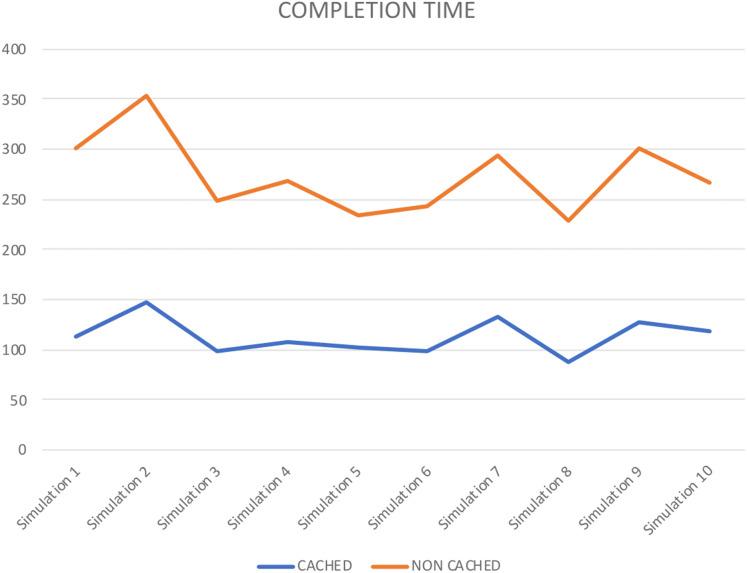
Completion time.

Figure 12 also represents the completion time for each simulation performed. It shows that in each simulation, cached data takes less time than non-cached. Therefore, it shows that the proposed approach improves storage resource utilization. Further, it validates the precision of our approach.

## Conclusion

This research is about the issues of data storage and retrieval from cloud-based data centers. Storage cost and bandwidth latency are the two major factors that influence the performance of a system. To reduce the bandwidth latency, most cloud service providers are using multiple copies of data, each on a separate data center across the world. Moreover, data centers are expensive to build and also are unfriendly to the environment. Erasure codes are the techniques that store data of *n* chunks in *n + k* data places. However, erasure codes need some extra computation time to regenerate the data. CAROM combined both techniques for dual benefits.

This research formally modeled CAROM using CPN formalism. Furthermore, we formally verified our model with space state analysis. Moreover, we formally analyzed the performance of our model by performing several simulations using monitors in CPN-Tools. Performance reports generated by CPN-Tools show that the model outperforms the others. In the presented model, the cache size is fixed. The cache is replaced by using the Least Frequently Used replacement algorithm. In the future, we will use some heuristic algorithms to resize and replace the cache in cloud-based systems.

## Supplemental Information

10.7717/peerj-cs.351/supp-1 Supplemental Information 1 Simulation code used to check model.

## Additional Information and Declarations

### Data Availability

The following information was supplied regarding data availability: Raw data are available as a Supplemental File.
