# Supplementary material for: Petri Net based modeling and analysis for improved resource utilization in cloud computing
Source: PeerJ Comput Sci. 2021 Feb 8;7:e351. doi: 10.7717/peerj-cs.351 (PMC7959626; doi:10.7717/peerj-cs.351)
Supplement: Supplemental Information 1 [file peerj-cs-07-351-s001.zip › Best Run Simulation Result 2/output/PerfReport.html]

CPN Tools Simulation Performance Report

CPN Tools Simulation Performance Report  
Net: /cygdrive/E/Office PC/SD Card/DROPBOX/Best Run Simulation Result/thesis -7.cpn

---

Note that these statistics have been calculated for data that is not necessarily independent or identically distributed.

| Timed statistics | | | | |
| --- | --- | --- | --- | --- |
| Name | Count | Avrg | Min | Max |
| Marking\_size\_Cache'Cache\_1 | 19 | 1.000000 | 0 | 1 |
| Marking\_size\_DB1'DB1\_1 | 7 | 1.000000 | 1 | 1 |
| Marking\_size\_DB2'DB2\_1 | 6 | 1.000000 | 1 | 1 |
| Marking\_size\_DB3'DB3\_1 | 8 | 1.000000 | 1 | 1 |
| Marking\_size\_Network'CacheHit\_1 | 6 | 0.087566 | 0 | 1 |
| Marking\_size\_Network'Response\_1 | 4 | 0.581436 | 0 | 1 |
| Marking\_size\_Network'Send\_Queue\_1 | 5 | 0.553415 | 0 | 1 |
| Marking\_size\_Store'Split\_\_1 | 10 | 0.000000 | 0 | 1 |

| Untimed statistics | | | | | |
| --- | --- | --- | --- | --- | --- |
| Name | Count | Sum | Avrg | Min | Max |
| Count\_trans\_occur\_Cache'Cache\_Checked\_1 | 3 | 3 | 1.000000 | 1 | 1 |
| Count\_trans\_occur\_Cache'Cache\_Full\_1 | 4 | 4 | 1.000000 | 1 | 1 |
| Count\_trans\_occur\_Cache'Remove\_LRU\_1 | 4 | 4 | 1.000000 | 1 | 1 |
| Count\_trans\_occur\_Cache'Store\_in\_Cache\_1 | 6 | 6 | 1.000000 | 1 | 1 |
| Count\_trans\_occur\_DB1'GetData\_1 | 1 | 1 | 1.000000 | 1 | 1 |
| Count\_trans\_occur\_DB2'Get\_Data2\_1 | 1 | 1 | 1.000000 | 1 | 1 |
| Count\_trans\_occur\_DB3'Get\_Data3\_1 | 1 | 1 | 1.000000 | 1 | 1 |
| Count\_trans\_occur\_Network'Receive\_1 | 2 | 2 | 1.000000 | 1 | 1 |
| Count\_trans\_occur\_Network'Receive\_Ack\_1 | 2 | 2 | 1.000000 | 1 | 1 |
| Count\_trans\_occur\_Network'Send1\_1 | 2 | 2 | 1.000000 | 1 | 1 |
| Count\_trans\_occur\_Network'Transmit\_Ack\_1 | 2 | 2 | 1.000000 | 1 | 1 |
| Count\_trans\_occur\_Network'Transmit\_Data\_1 | 2 | 2 | 1.000000 | 1 | 1 |
| Count\_trans\_occur\_ReGenerate'Reg\_Data\_1 | 0 | 0 | 0.000000 | 0 | 0 |
| Count\_trans\_occur\_ReGenerate'Send2\_1 | 0 | 0 | 0.000000 | 0 | 0 |
| Count\_trans\_occur\_Store'Split\_Data\_1 | 4 | 4 | 1.000000 | 1 | 1 |
| ReceiveData | 2 | 574 | 287.000000 | 239 | 335 |
| SendData | 2 | 528 | 264.000000 | 214 | 314 |
| Send\_Cache\_Data | 1 | 129 | 129.000000 | 129 | 129 |
| Send\_Regenerated\_Data | 0 | 0 | 0.000000 | 0 | 0 |
| Transmit\_ACK | 2 | 691 | 345.500000 | 279 | 412 |
| Transmit\_Data | 2 | 528 | 264.000000 | 214 | 314 |

Simulation steps executed: 63  
Model time: 571

---

Generated: Tue Feb 12 13:22:33 2019
